# Medical Mistrust in Online Cancer Communities: A Large‐Scale Analysis Across 10 Cancer Entities

**DOI:** 10.1002/pon.70180

**Published:** 2025-05-31

**Authors:** Fabian von Bubnoff, Johannes Werner, Nils R. Hebach, Deepti A. Chopra, Marc Cicero Schubert

**Affiliations:** ^1^ Medical Faculty Heidelberg Heidelberg University Heidelberg Germany; ^2^ Division of Cancer Medicine The University of Texas MD Anderson Cancer Center Houston Texas USA; ^3^ Department of Psychiatry The University of Texas MD Anderson Cancer Center Houston Texas USA

**Keywords:** artificial intelligence, cancer, large language models, medical mistrust, mistrust in oncology, oncology, patient trust, psychiatry, social media

## Abstract

**Background:**

Medical mistrust is a barrier to optimal cancer care. Analyzing social media posts where patients voice mistrust provides an opportunity to understand its variations and derive potential ways to address medical mistrust.

**Aims:**

To (1) identify the frequency of mistrust expression in cancer‐related Reddit posts, (2) characterize mistrusted entities and reasons for mistrust, and (3) identify emotional tone associated with mistrust.

**Methods:**

101,963 posts from 10 entity‐specific cancer communities on the social media platform Reddit made before September 30, 2024, were analyzed using a Large Language Model (LLM, “gpt‐4o‐mini”) in this cross‐sectional study. Performance of the LLM was compared to human raters. Categories for mistrusted entities and reasons for mistrust were developed inductively by human evaluators. Subsequently, posts were assigned to these different categories by the LLM.

**Results:**

Of *n* = 101,963 posts analyzed, 19,159 posts (18.8%) were categorized as expressing mistrust, predominantly directed at healthcare professionals (*n* = 14,221, 74.2%). Most common reasons for mistrust were “disregard for patient concerns” (*n* = 8176, 42.7%), “perceived incompetence of medical management” (*n* = 4871, 25.4%), and problems in “communication” (*n* = 4060, 21.2%). Mistrust posts commonly contained “worried” (*n* = 5933, 31.0%), “concerned” (*n* = 3623, 18.9%) and “frustrated” (*n* = 3046, 15.9%) tones.

**Conclusions:**

Expression of medical mistrust is prevalent in social media and is predominantly directed at healthcare professionals. Mistrust is frequently associated with dismissal of patients' symptoms or concerns, a perceived lack of thoroughness in clinical management and communication difficulties, suggesting these as key actionable areas to address medical mistrust in clinical practice.

## 1 Background

Cancer is one of the major public health problems of modern society [1]. A cancer diagnosis imposes a significant psychosocial burden on patients and relatives [2], making a strong physician‐patient relationship essential [3]. At the same time, a large proportion of cancer patients exhibit unmet supportive care needs and trust of these patients in healthcare professionals and institutions plays an important role in the appropriate diagnosis and treatment of cancer [4, 5]. For instance, the high prevalence of mistrust toward the health care system was reported to contribute to a lower participation in breast, prostate or colorectal cancer screening in marginalized groups [6, 7, 8, 9, 10]. Understanding why patients mistrust the healthcare system is critical to address these concerns in clinical practice [11].

Researchers have pursued different methodological approaches to examine medical mistrust among patients. For instance, questionnaires like the group‐based medical mistrust scale (GBMMS) and the LaVeist medical mistrust index or the trust in oncologist scale were developed to quantitatively measure medical mistrust in select patient groups and individuals, respectively [12, 13, 14, 15, 16]. Other approaches to analyze medical mistrust include patient interviews, providing insights into the specific reasons for mistrust toward healthcare [9, 17]. While the acquisition of data in a controlled setting constitutes a major benefit of these methods, it may provide an incomplete picture of the medical mistrust expressed by patients, as patients might withhold their concerns due to potential skepticism toward investigators. Furthermore, there is a risk of selection bias, as patients with a high degree of medical mistrust may be less likely to participate in questionnaire‐ or interview‐based research on this topic. In this study, we sought to comprehensively explore medical mistrust of Reddit users in cancer communities on Reddit. Assessing individual thoughts of cancer patients and their relatives about healthcare actors in a real‐world setting could provide valuable insights into the causes and potential solutions to medical mistrust.

The advent of Large Language Models (LLMs) makes analyses of text data increasingly feasible, for instance to extract structured information [18] or emotional tones from texts [19]. Recent research employed LLMs to explore specific questions in various fields of medicine [20, 21, 22]. In this study, we leverage a LLM to analyze medical mistrust in a cohort of more than 100,000 social media posts about cancer. With this analysis, we aim to estimate the prevalence of mistrust in cancer‐related social media posts and to identify the mistrusted entities as well as specific reasons for mistrust. Finally, medical mistrust can be associated with a variety of emotional tones including anxiety and frustration [23, 24, 25]. To fully understand the dimensions of medical mistrust in our study cohort, we sought to identify the language tone factors associated with the expression of mistrust.

## 2 Methods

### 2.1 Dataset and Preprocessing

Reddit is a social media platform organized into user‐created discussion boards called “subreddits”, which are communities focused on specific topics, allowing people to share experiences, ask questions, and engage with others who share similar interests [26]. On medicine‐related subreddits, patients and healthcare professionals share their questions, concerns, and opinions. Reddit data, which is publicly available, has been harnessed to analyze the needs and concerns of patients with several cancers [27, 28, 29]. These analyses mostly focused on the in‐depth analysis of a limited number of posts. The quantitative analysis of a large number of Reddit posts provides real‐world insights into the feelings, problems and expectations among patients with a variety of cancer diagnoses.

**FIGURE 1:**
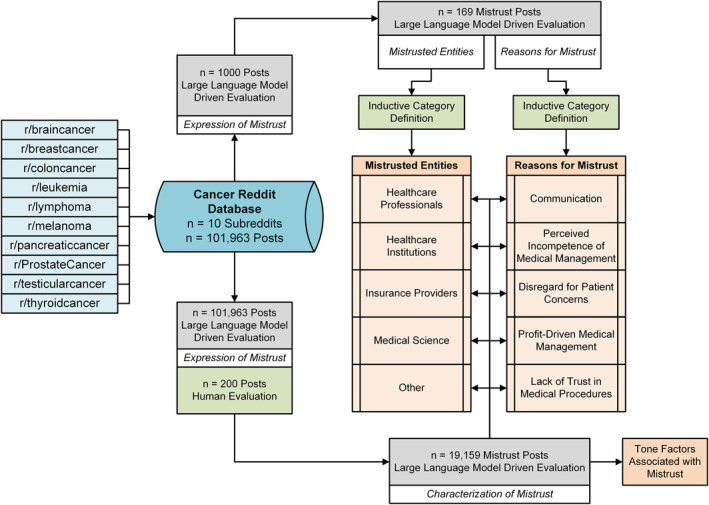
Analysis workflow for analyzing mistrust in Reddit posts across cancer‐specific subreddits.

**FIGURE 2:**
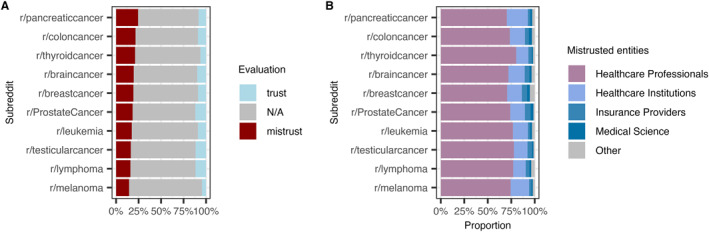
(A) Percentage of posts containing mistrust across subreddits. (B) Mistrusted entities across subreddits.

**FIGURE 3:**
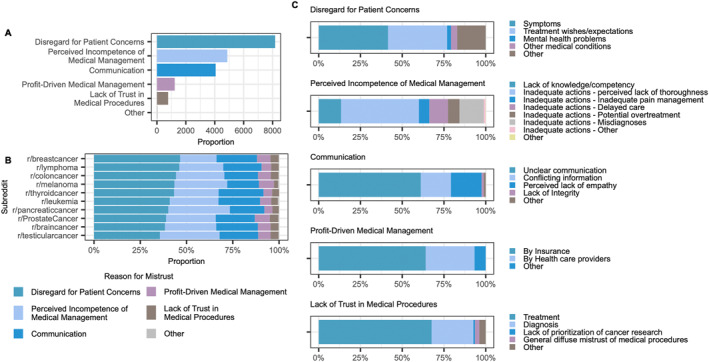
(A) Number of posts per reason category. (B) Distribution of reasons for mistrust by subreddits. (C) Granular reason analysis for all main reason categories identified.

**FIGURE 4:**
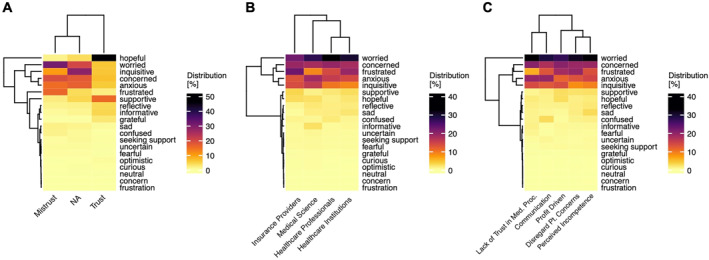
Tone analysis. (A) Tone analysis across trust categories. (B) Across mistrusted entities. (C) Across reasons for mistrust.

Using the publicly available aggregated subreddit metadata from the Project Arctic Shift archive [30], potentially cancer‐related subreddits were identified by name keyword filtering (“cancer”, “carcinoma”, “melanoma”, “sarcoma”, “lymphoma”, “oncology”, “tumor”, “neoplasm”, “metastasis”, “leukemia”, “glioma”, “mesothelioma”, “adenoma”, “myeloma”, “colorectal”). The ten entity‐specific subreddits containing the largest number of posts were selected. Using the Project Arctic Shift download tool [30], content and metadata of all posts posted in these subreddits before September 30, 2024, were downloaded, which resulted in *n* = 248,837 posts. Empty, removed, deleted, or posts with a length of less than 50 characters were excluded. Additionally, posts made by moderators and administrators were excluded. The final dataset consisted of *n* = 101,963 posts and was used for all subsequent analyses (Figure 1, see further dataset characteristics in Supporting Information S1: Figures 1–4).

### 2.2 Model Used in This Study

The LLM “gpt‐4o‐mini” was accessed via the OpenAI‐Application Programming Interface using the programming language Python.

### 2.3 Definition of Mistrust

Medical mistrust has been variably defined in the literature, often reflecting different conceptual focuses. These definitions encompass the belief that healthcare professionals or institutions do not act in the best interest of patients, especially if patients are part of a marginalized community [31, 32, 33, 34]. Existing literature conceptualizes mistrust as more than the absence of trust, but as an expectation of harm or neglect, often shaped by historical injustices, institutional failures, and group‐based experiences of discrimination [14]. It can be directed at individual healthcare professionals, healthcare organizations, or the medical system in general, influencing patient behaviors and healthcare engagement. The GBMMS recognizes mistrust as both an individual and collective experience, emphasizing suspicion, perceived disparities, and lack of support [35].

In this study, we sought to operationalize medical mistrust in a broad yet precise manner, ensuring to capture both systemic and interpersonal dimensions of mistrust. Medical mistrust was therefore defined as the belief that healthcare systems, institutions, or professionals may intentionally or unintentionally cause harm, discriminate, or fail to act in the patient's best interest. This definition was provided to the LLM explicitly.

### 2.4 Definition of Categories for Mistrust Reasons and Mistrusted Entities

In an exploratory analysis, *n* = 1000 posts were randomly sampled and subjected to the LLM for evaluation of specific mistrusted entities and reasons for mistrust. The LLM reported *n* = 169 different mistrust reasons (Supporting Information S1: Table 1). Human evaluators distilled these raw categories into unique, non‐overlapping categories in a stepwise process guided by Braun and Clarke's thematic analysis [36] (Supporting Information S1: Figure 5). First, the evaluators reviewed the raw categories multiple times to become familiar with the content of the raw categories. Similar categories were then grouped into initial themes, which were then refined into the final categories through several reiterations of the process. Through this analysis, the posts were grouped into the following main categories of reasons for mistrust: Communication (*n* = 53), Perceived Incompetence of Medical Management (*n* = 46), Disregard for Patient Concerns (*n* = 25), Lack of Trust in Medical Procedures (*n* = 18), Profit‐Driven Medical Management (*n* = 12) and Other (*n* = 15). For each main category, sub‐categories were inductively developed using the same method (Supporting Information S1: Figure 6, Supporting Information S1: Table 1), as were mistrusted entities. The final categories and subcategories were provided to the LLM as options to choose from for the scoring of the whole dataset. Exemplary Reddit posts and LLM‐assigned categories are shown in Supporting Information S1: Table 2.

### 2.5 Measurement of Mistrust, Mistrusted Entities and Reasons of Mistrust

To assess the expression of mistrust, we subjected the title and text of each post in our dataset to analysis by the LLM. In the prompt, we specified the desired output to classify a post as expressing mistrust, trust or neither of both. Furthermore, the prompt specified to describe the mistrusted entities and reasons of mistrust in the posts classified as “mistrust”, both as free text and according to pre‐specified categories. The exact prompt wording is available in the Supplementary Material File (Supporting Information S1: Supplementary Methods).

To determine the accuracy of the LLM‐based text analysis and classification, human raters (*n* = 3) assessed the expression of mistrust in *n* = 200 posts randomly sampled from the complete dataset. Raters were blinded to the AI evaluation and each other. The majority vote of the three human raters was considered gold standard and used for evaluation of the LLM ratings. The interrater‐reliability of the human evaluators was moderate (Fleiss' kappa: 0.46), indicating the subjectivity of mistrust inference from short snippets of text. Sensitivity and specificity for mistrust detection by the LLM compared to human consensus was 0.67 and 0.92, respectively, with an F1 score of 0.64. The specific prompts used to instruct the LLM are provided in the Supplementary Material File (Supporting Information S1: Supplementary Methods).

### 2.6 Tone Definition

In the exploratory analysis of *n* = 1000 posts, the LLM was instructed to return specific terms describing the tone of the text. Initially, the *n* = 10 most frequent occurring terms were used as categories to choose from for the LLM on the whole dataset (anxious, concerned, inquisitive, hopeful, worried, frustrated, uncertain, informative, supportive, curious), and subsequently, the top *n* = 20 terms were used as categories to choose from for the LLM. These were used for statistical analyses. A full list of identified tones can be found in Supporting Information S1: Table 3.

### 2.7 Reporting Guidelines

This study follows the Minimum Information for Medical AI Reporting framework reporting guidelines [37]. Additionally, for the qualitative aspects of our study regarding category definition, we report the relevant portions of the Consolidated Criteria for Reporting Qualitative Research guidelines [38].

### 2.8 Statistical Analyses

Analysis was performed using R (version 4.3.1) and Python (versions 3.11.8 and 3.12.2). Chi‐Squared test was used to test for significant differences regarding observed frequencies of variables between groups when comparing frequencies between subreddits, between mistrust reasons and mistrusted entities. Furthermore, the Chi‐Squared test was used to compare frequencies of tones used in post across the categories mentioned above, respectively. Cramér's V was determined to assess the strength of association of categorical variables. For all analyses, significance level was set to *p* = 0.05. Bonferroni correction was used to adjust for multiple testing. *p* values < 0.001 were reported as such.

## 3 Results

A total of *n* = 101,963 posts across the 10 largest cancer specific subreddits were evaluated by the LLM and labeled as either containing expressions of mistrust, expressions of trust, or neutral/lacking sufficient information to infer trust or mistrust (Figure 1). According to the LLM‐driven evaluation, 19,159 posts (18.8%) contained expressions of mistrust, 9315 posts (9.1%) contained expressions of trust, while the remaining 73,489 posts (72.1%) were neutral/lacking sufficient information.

The percentage of posts expressing mistrust differed significantly between subreddits (*p* < 0.001, Chi‐Squared‐Test, Figure 2A), with moderate absolute differences. Subreddits about pancreatic cancer, colon cancer and thyroid cancer contained higher percentages of mistrust posts (24.5%, 21.6%, 21.2%, respectively), with mistrust posts representing a smaller fraction of posts in melanoma (14.5%), lymphoma (16.0%), and testicular cancer (16.4%) subreddits. The breast cancer subreddit, which was the largest subreddit by total posts (Supporting Information S1: Figure 1), contained 19.6% mistrust posts.

To better understand the expressed mistrust, we sought to investigate mistrusted entities and underlying reasons for mistrust. In an unbiased fashion, *n* = 1000 posts were randomly sampled, and posts containing mistrust were categorized by the LLM whether mistrust is expressed, and if so with regards to entities and underlying reasons. We summarized the output of the LLM into five distinct categories of mistrusted entities, and five broad categories of underlying reasons. As specific mistrusted entities, we identified “Healthcare Professionals”, “Healthcare Institutions”, “Insurance Providers”, “Medical Science” and “Other”. As common reasons for mistrust, we identified “Communication”, “Disregard for Patient Concerns”, “Perceived Incompetence of Medical Management”, “Profit‐Driven Medical Management”, or “Lack of Trust in Medical Procedures”. Examples for all the categories are shown in Table 1. For reasons of mistrust, more granular categories were defined for further analyses (Supporting Information S1: Figure 5, Supporting Information S1: Table 2).

**TABLE 1:** Frequency of emotional tones across posts (trust, mistrust, or neutral/insufficient information) as assessed by the LLM.

Tone	Mistrust	NA	Trust	Chi‐squared stat	Adjusted *p* value	Cramér's V
Inquisitive	1915 (10)	20,828 (28.3)	563 (6)	4544.717	< 0.0001	0.2111
Worried	5933 (31)	14,678 (20)	559 (6)	2474.245	< 0.0001	0.1558
Concerned	3623 (18.9)	12,618 (17.2)	620 (6.7)	758.4785	< 0.0001	0.08625
Anxious	2870 (15)	12,160 (16.5)	520 (5.6)	770.3274	< 0.0001	0.08692
Hopeful	261 (1.4)	2746 (3.7)	4276 (45.9)	23,351.24	< 0.0001	0.4786
Frustrated	3046 (15.9)	2883 (3.9)	171 (1.8)	4188.079	< 0.0001	0.2027
Supportive	384 (2)	3105 (4.2)	1434 (15.4)	2653.851	< 0.0001	0.1613
Reflective	255 (1.3)	1120 (1.5)	382 (4.1)	345.576	< 0.0001	0.05822
Informative	94 (0.5)	751 (1)	400 (4.3)	838.2484	< 0.0001	0.09067
Sad	287 (1.5)	830 (1.1)	41 (0.4)	62.55687	< 0.0001	0.02477
Confused	262 (1.4)	471 (0.6)	9 (0.1)	167.5702	< 0.0001	0.04054
Seeking support	65 (0.3)	523 (0.7)	21 (0.2)	59.37622	< 0.0001	0.02413
Grateful	23 (0.1)	84 (0.1)	243 (2.6)	1537.992	< 0.0001	0.1228
Uncertain	68 (0.4)	265 (0.4)	15 (0.2)	9.809502	0.44	0.009809
Fearful	58 (0.3)	211 (0.3)	6 (0.1)	16.20095	0.02	0.01261
Curious	7 (0)	126 (0.2)	14 (0.2)	19.24296	0	0.01374
Optimistic	3 (0)	23 (0)	34 (0.4)	164.0185	< 0.0001	0.04011
Neutral	2 (0)	47 (0.1)	7 (0.1)	8.693295	0.76	0.009234
Frustration	1 (0)	10 (0)	0 (0)	2.097392	> 0.99	0.004535
Concern	2 (0)	8 (0)	0 (0)	1.008639	> 0.99	0.003145

*Note:* Chi‐squared test and Cramér’s V were computed; *p*‐values were adjusted using the Bonferroni correction.

In the majority of posts containing mistrust, the LLM‐evaluation showed that the mistrust was directed toward Healthcare Professionals (74.2%, *n* = 14,221), followed by mistrust toward Healthcare Institutions (15.9%, *n* = 3054), Insurance Providers (4.3%, *n* = 823), Medical Science (2.4%, *n* = 468) or scored as Other (3.1%, *n* = 593), with similar proportions across subreddits (Figure 2B). High‐granularity subanalysis of Healthcare Professionals and Other mistrusted entities is shown in Supporting Information S1: Figures 7 and 8.

Reasons for mistrust were most commonly categorized as Disregard for Patient Concerns (42.7%, *n* = 8176), followed by Perceived Incompetence of Medical Management (25.4%, *n* = 4871) and problems with Communication (21.2%, *n* = 4060) (Figure 3A). Significant differences were identified between cancer‐specific subreddits (*p* < 0.001, Chi‐Squared‐Test), with Disregard for Patient Concerns as the most common reason for mistrust across subreddits (Figure 3B).

To characterize the reasons for mistrust more granularly, the LLM was tasked to categorize posts into one of the specific subcategories defined before (Figure 3C). In posts with mistrust labeled as caused by “Disregard for Patient Concerns”, posts most often reported concerns regarding dismissed symptoms (41.5%, *n* = 3396 of 8176 posts) and dismissed treatment wishes (35.4%, *n* = 2891 of 8176 posts).

A perceived lack of thoroughness was the most common reason among posts where mistrust was scored as caused by “Perceived Incompetence of Medical Management” (46.6%, *n* = 2268 of 4871 posts), while unclear communication was the most common cause for mistrust across all posts where communication difficulties were labeled as the reason for mistrust (61.1%, *n* = 2482 of 4060 posts).

Lastly, we compared the emotional tone of posts according to the expression of trust and mistrust (Figure 4, Table 1).

This analysis revealed significant differences in the proportion of emotional tones between posts expressing trust and mistrust. For instance, a hopeful tone was strongly associated (Cramér's V = 0.4786, adjusted *p*‐value < 0.0001) with posts expressing trust (45.9%, *n* = 4276 of 9315 posts) compared to posts expressing mistrust (1.9%, 58 of 19,159 posts) or neutral posts (3.7%, 2746 of 73,489 posts). Posts with a frustrated tone were associated (Cramér's V = 0.2027, adjusted *p*‐value < 0.0001) with the expression of mistrust (19.2%, *n* = 3697 of 19,159 posts) compared to the expression of trust (1.8%, 171 of 9315 posts) or neutral posts (3.9%, 2883 of 73,489 posts). An inquisitive tone was associated (Cramér's V = 0.2111, adjusted *p*‐value < 0.0001) with neutral posts (28.3%, 20,828 of 73,489 posts) compared to posts expressing mistrust (10.0%, 1915 of 19,159 posts) or trust (6%, 563 of 9315 posts) (Figure 4A).

Furthermore, we analyzed the tones of posts expressing mistrust according to the mistrusted entity and the specific reason for mistrust. This analysis reveals significant differences with minor effect sizes (Cramér's V < 0.1) of tones between the mistrusted entities, while the most frequent tones observed in posts expressing mistrust are well reflected across all mistrusted entities (Figure 4B, Supporting Information S1: Table 4). When comparing the tone of posts expressing mistrust according to the reason for mistrust, we observed a high proportion of worried and concerned posts across all reasons for mistrust (Figure 4C, Supporting Information S1: Table 5). A frustrated tone frequently occurred in posts mentioning the disregard of patient concerns (19.5%, 1596 of 8176 posts) and profit‐driven medical management (18.4%, 227 of 1232 posts) compared to other reasons for mistrust (Cramér's V = 0.1011, adjusted *p*‐value < 0.0001). An anxious tone frequently occurred in posts mentioning communication issues (19.6%, 796 of 4060 posts) or the lack of trust in medical procedures (19.5%, 151 of 775 posts). Overall, the emotional tone of posts varies significantly between posts expressing trust or mistrust, but only to a minor extent according to the mistrusted entity and category of mistrust.

## 4 Discussion

This study constitutes a comprehensive real‐world characterization of medical mistrust, with more than 100,000 analyzed cancer‐related social media posts. In this dataset, we characterized common mistrusted entities and reasons of mistrust among patients and relatives across ten common cancer entities in a quantitative manner.

Previous research outlined mistrusted entities in the medical context as interpersonal, that is toward the healthcare professional, and systemic mistrust, that is toward the healthcare system, pharmaceutical companies, or political institutions [13, 33]. Our study found that in posts across all examined subreddits, frequently mistrusted entities were healthcare professionals, healthcare institutions, medical science and insurance companies. Prior studies have quantitatively outlined interpersonal and systemic mistrust in oncology patients in great detail [12, 39, 40, 41, 42]. However, since these studies have focused on structured surveys and recruited participants from healthcare settings, individuals who avoid care due to mistrust may potentially be underrepresented [12, 39, 40, 41, 42]. Our study leverages large‐scale computational analyses of patient discourse in online cancer communities, capturing the perspectives of those who may not engage with traditional healthcare research and offering new insights into the evolving dynamics of medical mistrust beyond clinical settings. Online social media platforms offer the opportunity to share experiences and receive feedback from online communities in an anonymous fashion, which allows patients to share thoughts that they might not share with a healthcare professional in person. Additionally, as this data is not acquired in a study setting but reflects the authors' concerns at the time of writing, a higher level of authenticity might be expected. Interestingly, interpersonal mistrust, mostly toward healthcare professionals, is the predominant category of mistrust and highly exceeds systemic mistrust in online cancer communities.

Studies investigating factors driving patients' trust in their healthcare professionals described different aspects of communication, such as honesty, patient prioritization and fidelity, but also their competence as important factors [11, 15, 17]. Our results show that mistrust toward healthcare professionals is driven by three major factors, namely the disregard for a patient's concerns in the patient‐provider interaction, the perceived medical incompetence of the healthcare professional, and communication.

Disregard for patients' symptoms or treatment expectations has been termed “medical gaslighting” in internet communities [43], and has previously been described as a reason for medical mistrust, for instance in the context of post‐COVID‐19 syndrome [44]. Our findings highlight that the perceived dismissal of medical concerns is also highly prevalent among cancer patients and is associated with the expression of frustration, thereby contributing to the development of mistrust toward the healthcare provider.

Systemic mistrust has often been described as overlapping with the belief in conspiracy theories and the suspicion of a medical system prioritizing financial profit over patient wellbeing [33]. This is partly reflected in our results, which identify “Profit‐driven Medical Management” as a major reason for medical mistrust. Our analysis demonstrates that this mistrust is attributed mainly to insurance providers and is expressed with a highly frustrated tone.

The analysis of tones associated with posts expressing mistrust revealed that mistrust is often accompanied by a worried, concerned, anxious or frustrated tone. Several studies investigating the emotional components of medical mistrust in chronic disease have described an association of mistrust and high levels of anxiety or frustration. For instance, medical mistrust was correlated to the level of anxiety in a cohort of black breast cancer patients [23]. Moreover, in African‐American men awaiting kidney transplant, mistrust of the transplantation process was linked to frustration toward physicians [24]. Further research showed that the mistrust of black patients with serious illnesses was characterized by high levels of suspicion and perceived lack of support [45], that medical mistrust often originated from perceived discrimination and was linked to anger, sadness as well as frustration in an indigenous population in Oregon [25]. Of note, these studies analyzed the emotional components of mistrust in specific communities. Our analysis confirms the frequent association of mistrust with anxious and frustrated tones, and contributes to the existing data that a relevant proportion of mistrust in cancer communities is associated with a worried and concerned tone.

Future research could build on these findings to explore mistrust in other cancer entities and chronic diseases than analyzed in this study. Considering that the number of patients sharing experiences and concerns online will most likely continue to rise, online communities might become an increasingly relevant source for collecting real‐world data to understand patients' needs.

### 4.1 Implications

The findings of this study indicate that social media can be used to systematically evaluate patients' medical mistrust and that medical mistrust is a prevalent issue among cancer patients engaging in online health communities. There is an urgent need to address medical mistrust in oncology, as trust in healthcare professionals is associated with better adherence to treatment, higher patient satisfaction, and improved quality of life [46], while mistrust has been linked to delays in seeking care, refusal of treatment, and avoidance of healthcare interactions [47].

The most frequently cited reason for mistrust in this study was disregard for patient concerns, especially dismissal of patient's symptoms and treatment preferences. Feeling unheard has been linked to poor adherence to medical recommendations and the pursuit of unreliable alternative information [48]. Providers can counteract this by actively listening, validating concerns, and engaging in shared decision‐making, potentially preventing adverse outcomes.

Furthermore, our analysis revealed that within the category of perceived incompetence‐based mistrust, lack of thoroughness was a key underlying factor. This is closely aligned with prior research demonstrating that even in the absence of diagnostic errors, incomplete investigations can erode patient trust [5]. In cases of rare cancers, it has been demonstrated that explicit confirmation of expertise and a detailed diagnostic workup are critical for establishing trust [49]. Clinicians should therefore allocate adequate consultation time, acknowledge uncertainties and clearly communicate the clinical reasoning process.

Communication issues were the third most prevalent mistrust reason with major underlying reasons being unclear communication, conflicting communication and lack of empathy. Conveying information in a comprehensible and consistent manner as well as incorporating established patient‐provider communication models like patient‐centered communication could help prevent and overcome communication difficulties [50]. Empathetic communication by oncologists not only builds trust but has also been shown to reduce patient anxiety, even when delivering difficult news [51].

Patients also expressed mistrust toward insurers when profit‐driven medical management was perceived, followed by mistrust toward healthcare professionals. Insurance‐based discrimination, especially among publicly insured or uninsured patients, drives mistrust, as these groups often experience denial of care or differential treatment [52, 53]. Moreover, when clinicians are seen as complicit with insurers, patients' mistrust deepens [54, 55]. To mitigate this, healthcare professionals can guide patients in understanding insurance constraints, advocate for necessary treatments, and explain cost limitations with empathy and transparency.

Awareness of the link between mistrust and specific emotional tones can help clinicians be more sensitive to detecting mistrust in patients. Since patients with mistrust are more likely to be worried, frustrated, concerned and anxious, clinicians can be attentive to signs of mistrust in those displaying a concerned emotional state and address these concerns early, thereby reducing potential negative outcomes associated with mistrust.

Additionally, as the prevalence of mistrust expression in social media posts was relatively high, healthcare professionals should be aware that individuals searching for patient experiences in online communities will likely be exposed to varying degrees of mistrust, which may influence their attitude toward cancer medicine as well as the patient‐provider relationship. Understanding this online landscape can help clinicians interpret mistrust, understand non‐patient‐provider interaction based mistrust and guide patients to reliable information if such concerns arise in clinical encounters.

### 4.2 Limitations

There are several limitations to this study. First, as a cross‐sectional study, our analyses are inherently descriptive. Second, as Reddit posts are anonymous, the identity as well as demographic, socioeconomic and ethnic information of a single user is unknown. In general, Reddit users are estimated to be predominantly young and male [56], which limits generalization of the findings to the general cancer patient population. Another limitation arising from the study cohort composition is that many studies investigating medical mistrust have focused on specific marginalized groups, possibly explaining the high proportion of interpersonal compared to systemic mistrust we observed. Additionally, validation of the LLM‐based mistrust classification model relies on subjective human ratings, since no established methods exist to definitely detect mistrust from short texts. While showing high specificity, our model exhibits only moderate sensitivity in a human‐labeled validation dataset. This could lead to underestimation of the actual mistrust frequency in Reddit posts. Moreover, only a subset of posts was validated by human evaluators, potentially resulting in classification errors. As the expression of mistrust was only approached as a categorical variable in this study, future work could integrate a linear scale to better analyze nuances of mistrust intensities. Finally, as mistrust was defined as described above, different results regarding frequency and categories of mistrust could have occurred when using different definitions of medical mistrust.

## 5 Conclusion

Our findings provide unprecedented quantitative insights into the reasons for and expression of medical mistrust, utilizing real world data of cancer patients in online communities. This information can help oncologists and other health care providers treating oncological patients to identify areas of improvement during patient interaction as well as create awareness for the heterogeneous social media landscape patients are exposed to. A relevant proportion of social media posts contained expressions of mistrust, most commonly directed toward healthcare professionals. Characterizing the heterogeneous reasons for mistrust revealed that disregard for patients' concerns and symptoms, perceived lack of thoroughness, and unclear communication are common reasons for mistrust. Tone analyses indicate that anxiety and worry is most strongly associated with mistrust. Overall, this study provides insights into the heterogeneity of mistrust in oncology across 10 entities and identifies actionable areas to address medical mistrust in clinical practice.

## Author Contributions

Conceptualization: F.v.B., J.W., N.R.H., D.A.C., M.C.S. Software Preprocessing: F.v.B. Software Large Language Model: M.C.S. Software Analysis: F.v.B., J.W., N.R.H., M.C.S. Inductive Analysis: J.W., M.C.S. Writing – Original Draft: J.W., M.C.S. Writing – Review and Editing: F.v.B., J.W., N.R.H., D.A.C., M.C.S.

## Ethics Statement

This study involved a secondary analysis of publicly available posts on social media. An ethics review is not required due to the public nature of the data and the absence of identifying information for the individuals creating the posts. Written informed consent was not applicable, as the study used archived, publicly available posts voluntarily shared in an open, public forum. Reddit's Privacy Policy states that by posting publicly on the platform, users agree to have their content shared publicly and freely. To minimize undue attention to specific posts, example posts included in the manuscript were modified in accordance with established guidelines [57].

## Conflicts of Interest

The authors declare no conflicts of interest.

## Supporting information

Supporting Information S1

## Data Availability

Software code used in this study and summary data tables analyzed from this study and can be accessed via https://github.com/schubertmc/LLMTrust. Archived reddit posts were accessed as described in methods above.
