# Supplementary material for: Medical Mistrust in Online Cancer Communities: A Large‐Scale Analysis Across 10 Cancer Entities
Source: Psychooncology. 2025 May 31;34(6):e70180. doi: 10.1002/pon.70180 (PMC12126172; doi:10.1002/pon.70180)
Supplement: Supplementary file 1 — Supporting Information S1 [file PON-34-e70180-s001.pdf]

Supplementary Material

Table of Contents

Supplementary Figure 1 2
Supplementary Figure 2 2
Supplementary Figure 3 3
Supplementary Figure 4 3
Supplementary Figure 5 4
Supplementary Figure 6 5
Supplementary Figure 7 6
Supplementary Figure 8 7
Supplementary Table 1 8
Supplementary Table 2 18
Supplementary Table 3 20
Supplementary Table 4 22
Supplementary Table 5 23
Supplementary Methods 24
Granular Categories for Reasons of Mistrust 24
Prompts Used in this Study 25
1. Exploratory post analysis 25
2. Main post analysis 25
3. Granular reason analysis 26
4. Post template 26
Category definition 27
Large Language Models for Information Extraction 27
References 29

Supplementary Figure 1

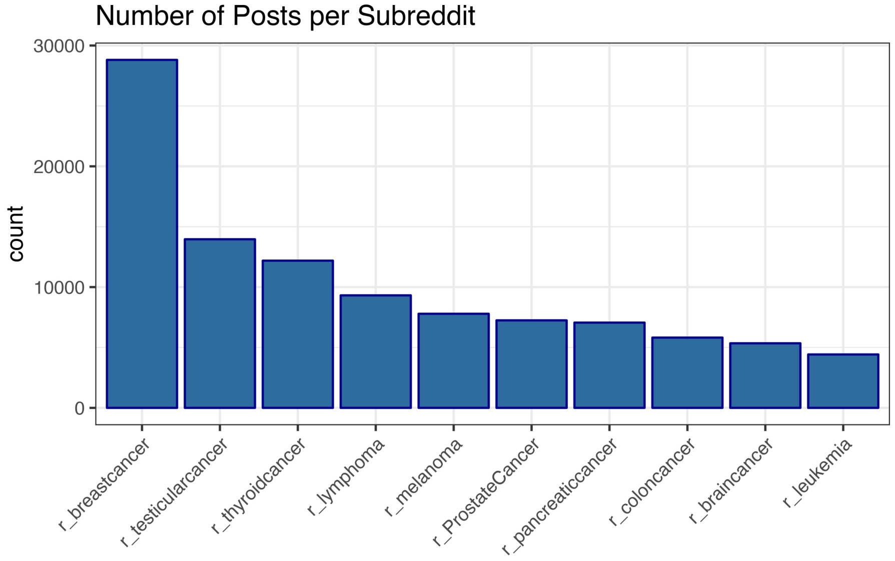

Supplementary Figure 1: Dataset characteristics - Number of posts per subreddit (n = 101,963 posts across n = 10 subreddits).

Supplementary Figure 2

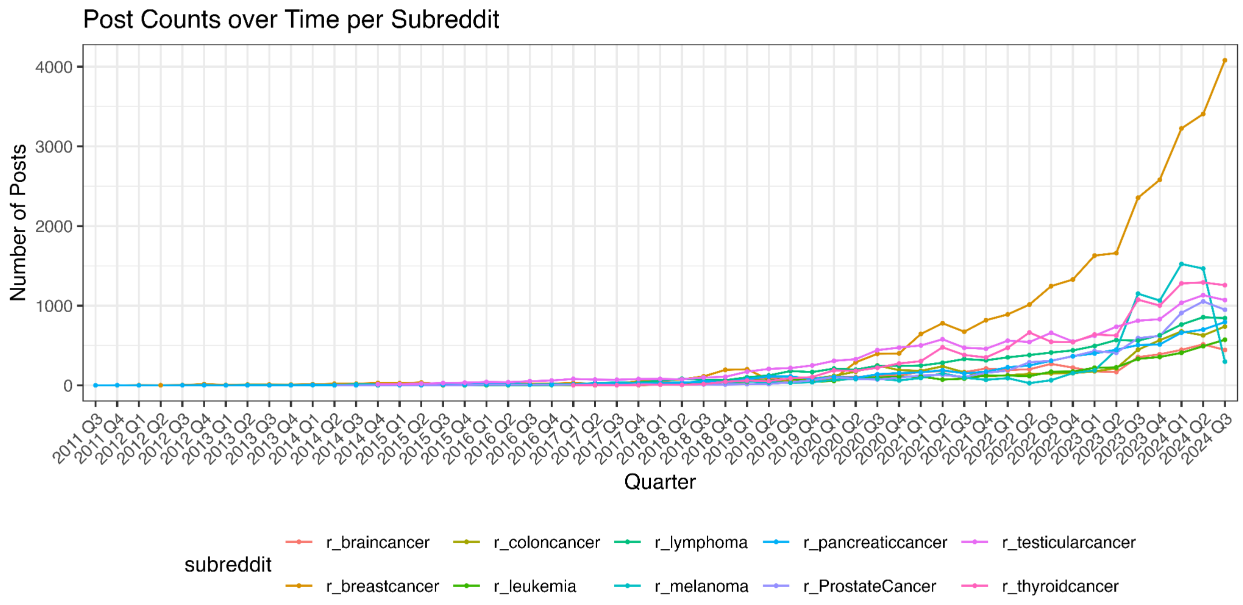

Supplementary Figure 2: Dataset characteristics - Number of posts per subreddit over time (n = 101963 posts).

Supplementary Figure 3

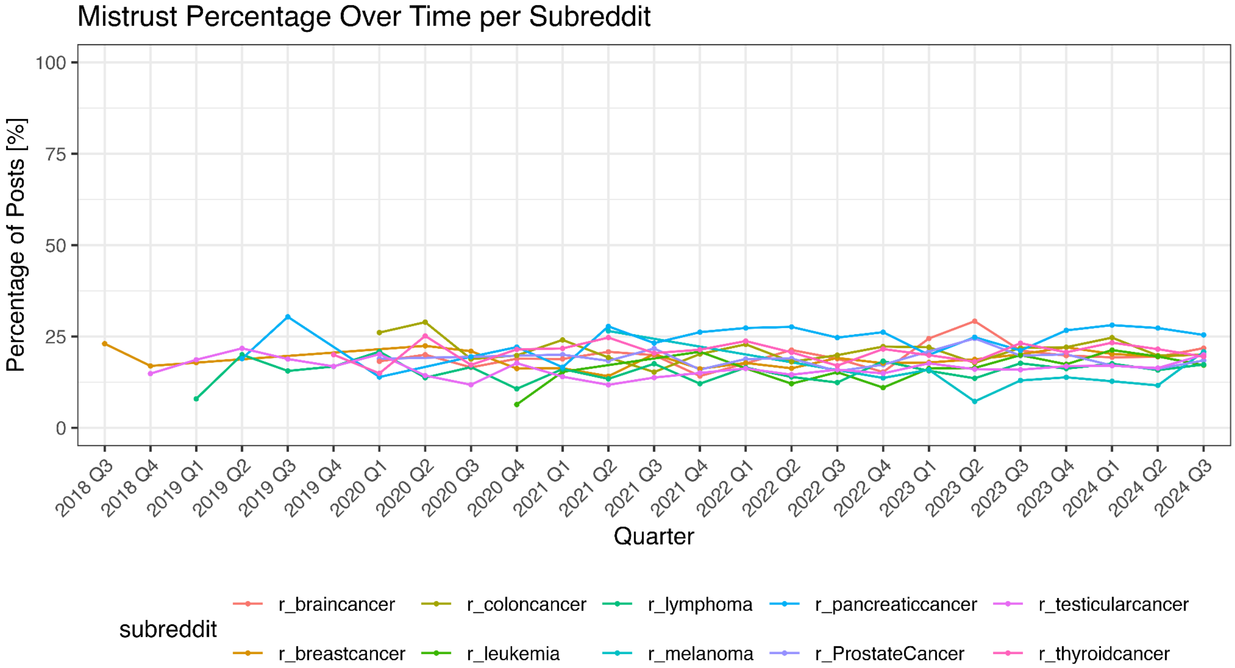

Supplementary Figure 3: Percentage of mistrust over time expressed over time (n = 101963 posts). Only quarters with more than 100 posts per quarter and subreddit are visualized in the analysis.

Supplementary Figure 4

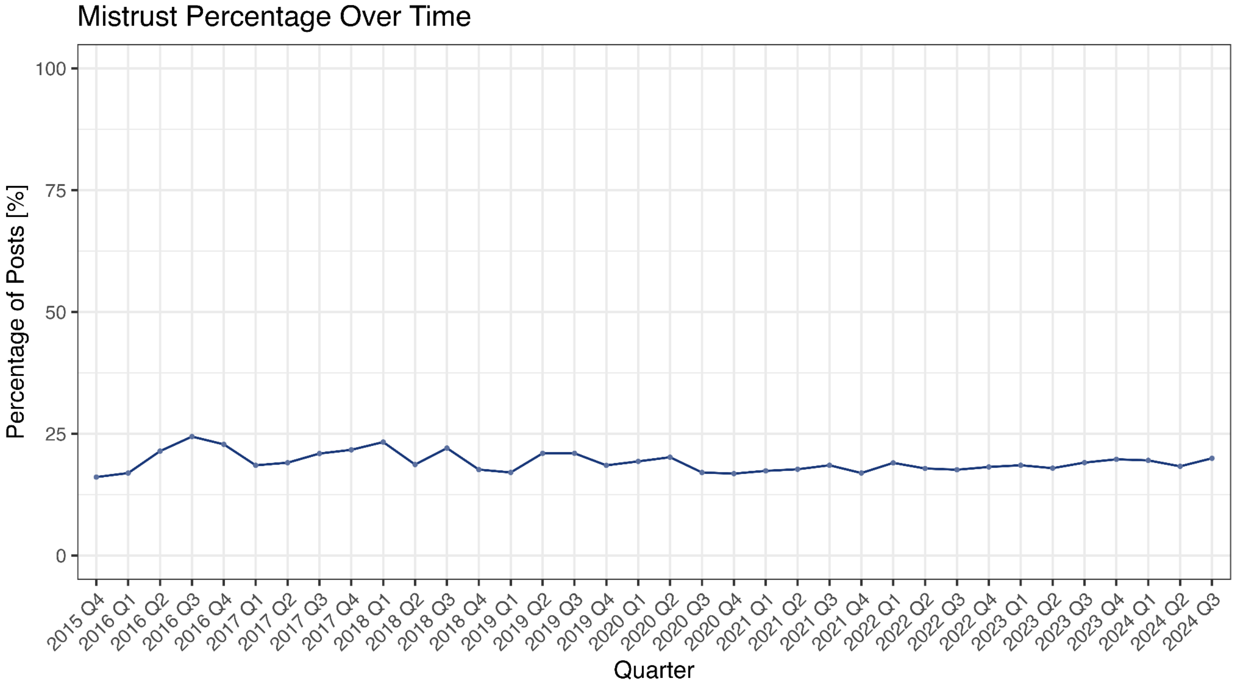

Supplementary Figure 4: Percentage of mistrust over time expressed over time (n = 101,963 posts). Only quarters with more than 100 posts per quarter are visualized in the analysis.

Supplementary Figure 5

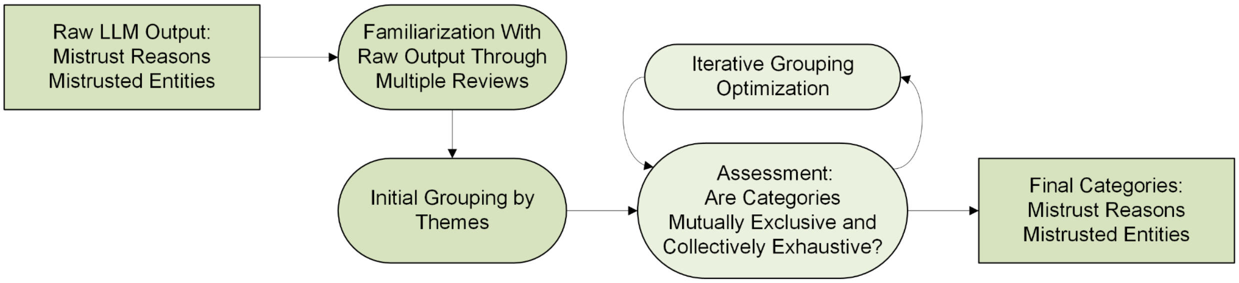

Supplementary Figure 5: Schematic of how categories were derived. After familiarization with the raw LLM output, categories identified by the LLM were iteratively grouped together to enhance analysis interpretability until they were mutually exclusive and collectively exhaustive.

Supplementary Figure 6

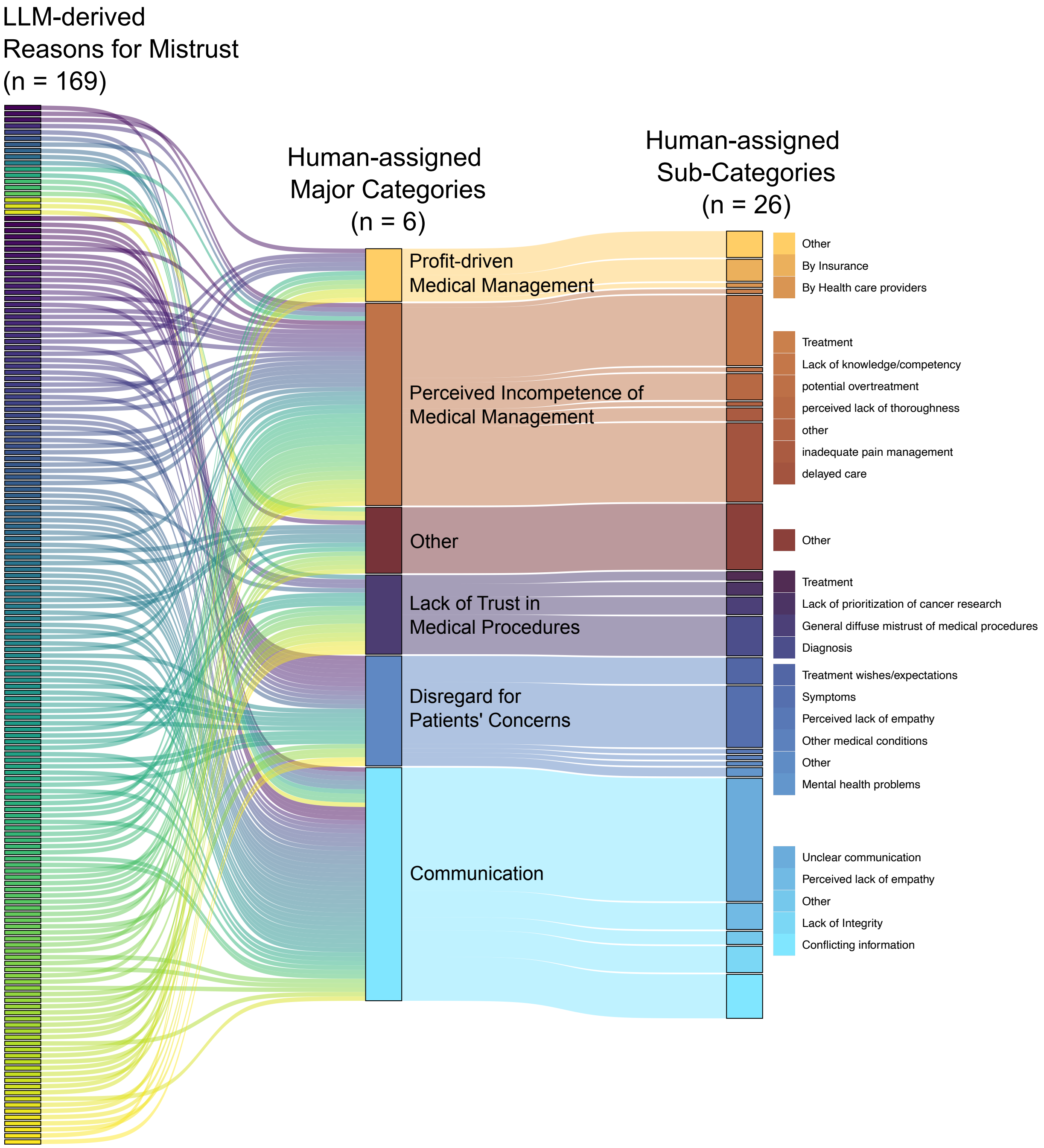

Supplementary Figure 6: Sankey Diagram of Mistrust Reason Categories as inductively developed by human evaluators.

Supplementary Figure 7

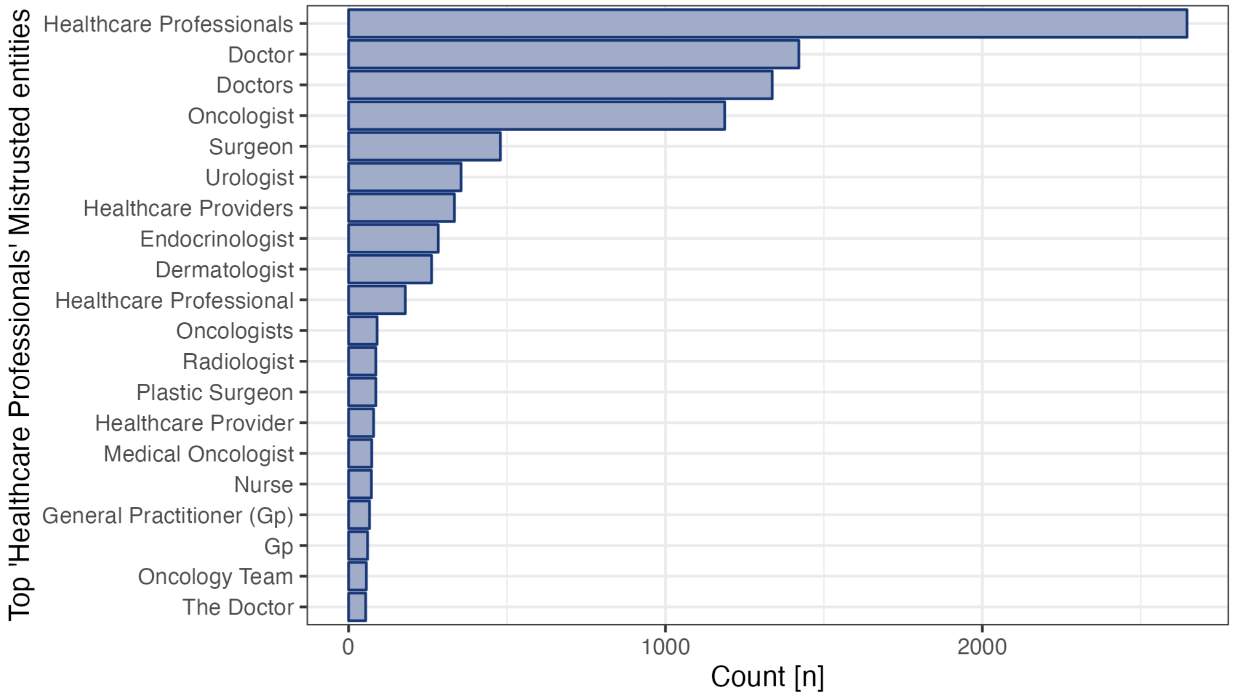

Supplementary Figure 7: Top 20 most often free text categories returned by the LLM when category ‘Healthcare Professionals’ was chosen as a mistrusted entity by the LLM (n = 14,217 posts, free text).

Supplementary Figure 8

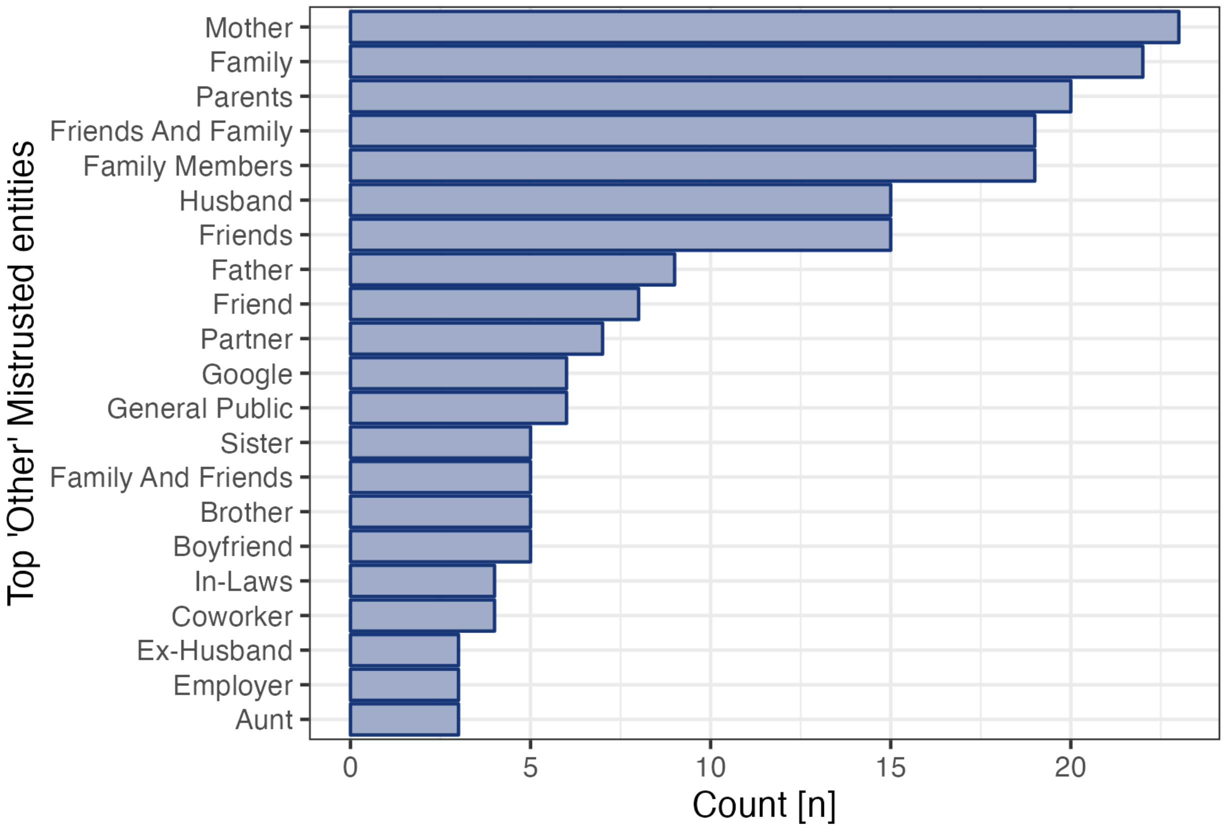

Supplementary Figure 8: Number of occurrences of free text mistrusted entities returned labeled as ‘Other’ that occurred at least 3 times, from n = 586 posts where the mistrusted entity was returned as ‘Other’.

Supplementary Table 1

Supplementary Table 1: Free text reasons extracted by the LLM in the discovery dataset, and the major and minor assigned categories for reasons of mistrust.

Post Number	Identified Reason by LLM	Major category assigned	Minor category assigned
1	Doubt about the efficacy or necessity of medical procedures (biopsy)	Lack of Trust in Medical Procedures	Diagnosis
2	Frustration with communication and treatment options provided by local healthcare professionals.	Communication	Unclear communication
3	lack of communication and information regarding the patient's condition and treatment	Communication	Unclear communication
4	Lack of communication and concern regarding increasing PSA levels.	Communication	Unclear communication
5	Delay in insurance approval process affecting timely treatment.	Profit-Driven Medical Management	By Insurance
6	Uncertainty about treatment decisions and the lack of explanation regarding the absence of ADT.	Communication	Unclear communication
7	Concerns about potential harm and complications from biopsy procedures.	Lack of Trust in Medical Procedures	Diagnosis
8	Frustration over receiving concerning medical information through apps instead of direct communication with a doctor.	Communication	Perceived lack of empathy
9	discrepancy in information about the presence of a pathologist during surgery	Communication	Conflicting information
10	Conflicting information about treatment efficacy	Communication	Conflicting information
11	Perceived dishonesty by healthcare providers	Communication	Lack of Integrity
12	Concern about the potential for harm due to the doctors' actions during the examinations.	Lack of Trust in Medical Procedures	Diagnosis
13	uncertainty about the necessity and safety of the proposed operation	Perceived Incompetence of Medical Management	Inadequate actions - potential overtreatment
14	Delays in treatment and testing based on insurance policies.	Profit-Driven Medical Management	By Insurance
15	Concerns about side effects leading to cognitive decline and depression.	Other	Other
16	Inability to contact customer service and potential issues with lab error resolution	Communication	Other
17	Concerns about the accuracy of the MRI equipment and communication from the medical staff.	Lack of Trust in Medical Procedures	Diagnosis
18	Concerns about misinformation.	Communication	Unclear communication

19	Lack of communication and feeling like a test case.	Communication	Lack of Integrity
20	Long wait time for biopsy and lack of communication from the healthcare office.	Communication	Other
21	trouble with insurance regarding the referral and evaluations	Profit-Driven Medical Management	By Insurance
22	Conflicting information regarding the diagnosis, leading to feelings of betrayal and uncertainty.	Communication	Conflicting information
23	Lack of clear communication and understanding from healthcare professionals regarding cancer prognosis and treatment.	Perceived Incompetence of Medical Management	Lack of knowledge/competency
24	Perceived lack of support in managing anxiety and sedation.	Perceived Incompetence of Medical Management	Inadequate actions - other
25	Previous misdiagnosis and current financial burden from healthcare costs.	Profit-Driven Medical Management	Other
26	Perceived delay and lack of decisive action regarding the biopsy and treatment.	Perceived Incompetence of Medical Management	Inadequate actions - delayed care
27	Lack of confidence in options due to rarity and limited studies available.	Lack of Trust in Medical Procedures	Lack of prioritization of cancer research
28	The post expresses frustration and guilt over the healthcare system's previous dismissal of symptoms, leading to a late diagnosis.	Disregard for Patient Concerns	Symptoms
29	Lack of guidance and support in navigating treatment options.	Communication	Unclear communication
30	Concern that healthcare professionals might not be fully supportive or effective in encouraging the patient's exercise.	Disregard for Patient Concerns	Treatment wishes/expectations
31	Perceived negligence in handling the referral process and treatment delays due to insurance issues.	Profit-Driven Medical Management	By Insurance
32	Concern about the long wait time for appointments and the anxiety it causes.	Perceived Incompetence of Medical Management	Inadequate actions - delayed care
33	Belief that the diagnostic procedure may have caused harm leading to cancer.	Lack of Trust in Medical Procedures	General diffuse mistrust of medical procedures
34	Inconsistent information and unclear communication regarding case presentation and treatment recommendations.	Communication	Conflicting information
35	Negligence in care leading to delays in treatment	Perceived Incompetence of Medical Management	Inadequate actions - delayed care
36	Belief that the doctor may have been incorrect in their assessment of the tumor's prognosis based on genetic markers.	Perceived Incompetence of Medical Management	Lack of knowledge/competency

37	Concerns about exploitation in hospice care	Profit-Driven Medical Management	Other
38	Father-in-law's negative past experience with a doctor (reaction to booster shot), leading to refusal of further medical care.	Lack of Trust in Medical Procedures	General diffuse mistrust of medical procedures
39	Perception that the healthcare system is not prioritizing cancer research and treatment despite advancements in other fields.	Lack of Trust in Medical Procedures	Lack of prioritization of cancer research
40	Perception of insufficient progress in finding a cure and personal losses in the family.	Lack of Trust in Medical Procedures	Lack of prioritization of cancer research
41	Desire to seek a second opinion and transfer to another hospital	Perceived Incompetence of Medical Management	Lack of knowledge/competency
42	Concerns about previous surgery complications and waiting for treatment.	Perceived Incompetence of Medical Management	Inadequate actions - delayed care
43	lack of clear information regarding reproductive options before treatment	Communication	Unclear communication
44	Uncertainty about diagnosis and potential neglect of symptoms over time.	Disregard for Patient Concerns	Symptoms
45	Perception of inadequate care and delayed access to specialists.	Perceived Incompetence of Medical Management	Inadequate actions - delayed care
46	general suspicion towards healthcare practices	Lack of Trust in Medical Procedures	General diffuse mistrust of medical procedures
47	The author expresses concern about their GP's lack of urgency or thoroughness regarding the mole's examination.	Perceived Incompetence of Medical Management	Inadequate actions - perceived lack of thoroughness
48	Previous dermatologists dismissed concerns despite noticeable changes.	Perceived Incompetence of Medical Management	Inadequate actions - perceived lack of thoroughness
49	Delay in dermatology appointment after expressing concerns about a changing mole.	Perceived Incompetence of Medical Management	Inadequate actions - delayed care
50	Concern over the possibility of missed diagnosis despite professional opinions	Lack of Trust in Medical Procedures	Diagnosis
51	Doubt about the dermatologist's assessment and lingering worry about health.	Perceived Incompetence of Medical Management	Lack of knowledge/competency
52	Perceived neglect of mental health support for cancer survivors.	Disregard for Patient Concerns	Mental health problems
53	Inconclusive biopsy results and unexpected post-operative issues.	Other	Other
54	Delayed care and lack of proper diagnosis leading to miscommunication.	Perceived Incompetence of Medical Management	Inadequate actions - delayed care

55	Concerns about the accuracy and timeliness of the initial diagnosis leading to complications.	Perceived Incompetence of Medical Management	Inadequate actions - delayed care
56	The author expresses frustration about previous misdiagnoses and lack of timely intervention by doctors.	Perceived Incompetence of Medical Management	Inadequate actions - delayed care
57	Concern that the oncologist may be running out of treatment options.	Other	Other
58	Feeling dismissed by healthcare providers regarding his concerns due to his age.	Disregard for Patient Concerns	Symptoms
59	Lack of clarity on the urgency and interpretation of scan results.	Communication	Unclear communication
60	Perceived lack of support and understanding of the difficulties faced post-treatment.	Communication	Perceived lack of empathy
61	Repeated dismissals of symptoms and reliance on diagnosis without thorough investigation.	Disregard for Patient Concerns	Symptoms
62	Assumption that their situation is good based on appearance, leading to disregard for the emotional and physical challenges faced.	Disregard for Patient Concerns	Mental health problems
63	conflicting information and lack of clear guidance from healthcare providers	Communication	Conflicting information
64	Concern about being treated like just another number and difficulty in getting appointments.	Profit-Driven Medical Management	Other
65	Skepticism about the accuracy of the surgeon's assessment regarding the cancer stage before all tests were completed.	Perceived Incompetence of Medical Management	Lack of knowledge/competency
66	Concerns about inadequate testing recommendations and family history.	Perceived Incompetence of Medical Management	Inadequate actions - perceived lack of thoroughness
67	The author feels pressured by the endocrinologist to try weight loss medications despite not wanting to, suggesting a lack of trust in her approach.	Disregard for Patient Concerns	Treatment wishes/expectations
68	Concerns about the indeterminate FNA results and repetition of tests leading to a potential delay in definitive treatment.	Perceived Incompetence of Medical Management	Lack of knowledge/competency
69	Concern about coverage for necessary procedures and treatment.	Profit-Driven Medical Management	By Insurance
70	Inability of the endocrinologist to provide a clear explanation or solution for the symptoms.	Perceived Incompetence of Medical Management	Lack of knowledge/competency
71	Inability to provide a conclusive diagnosis and the need for further evaluation by another institution.	Other	Other
72	Doctors' lack of attention to reported symptoms.	Disregard for Patient Concerns	Symptoms

73	The author expresses dissatisfaction with their previous PCP, implying a lack of care and concern.	Communication	Perceived lack of empathy
74	Frustration over the surgeon's recommendation against removable stitches.	Disregard for Patient Concerns	Treatment wishes/expectations
75	Uncertainty due to conflicting information about diagnosis and treatment.	Communication	Conflicting information
76	Concern about the adequacy of doctor's guidance regarding medication adjustment.	Perceived Incompetence of Medical Management	Lack of knowledge/competency
77	The use of the term 'difficult case' may suggest a lack of confidence in diagnosis.	Perceived Incompetence of Medical Management	Lack of knowledge/competency
78	Lack of communication about symptoms and concerns beyond the cancer diagnosis.	Communication	Unclear communication
79	dissatisfaction with pain management and ineffective medication	Perceived Incompetence of Medical Management	Inadequate actions - inadequate pain management
80	Concerns about previous overmedication and challenges in finding the right medication dosage.	Disregard for Patient Concerns	Treatment wishes/expectations
81	Perceived manipulation of treatment for profit and lack of consideration for patient well-being.	Profit-Driven Medical Management	By Health care providers
82	Concern over the accuracy and reliability of FNA results and the potential for misdiagnosis	Lack of Trust in Medical Procedures	Diagnosis
83	Feeling uninformed and confused about the surgery and follow-up care.	Communication	Unclear communication
84	Scheduling delays and impact on personal milestones.	Perceived Incompetence of Medical Management	Inadequate actions - delayed care
85	Perception that thyroid cancer is downplayed, leading to inadequate support	Disregard for Patient Concerns	Perceived lack of empathy
86	Lack of accountability and unclear communication from healthcare providers.	Communication	Unclear communication
87	Insufficient communication and support regarding dietary restrictions prior to RAI treatment.	Communication	Unclear communication
88	Belief that the vaccine may have triggered earlier detection of cancer.	Lack of Trust in Medical Procedures	General diffuse mistrust of medical procedures
89	Lack of communication and information about the low iodine diet.	Communication	Unclear communication
90	Concerns about pain and potential harm from the MRI procedure.	Lack of Trust in Medical Procedures	Diagnosis

91	Concern about the doctor's assumptions and accuracy of the previous examination results.	Perceived Incompetence of Medical Management	Lack of knowledge/competency
92	Being uninsured and concerns about access to medical care.	Profit-Driven Medical Management	Other
93	Doubt about the accuracy and clarity of medical information and decisions regarding treatment.	Communication	Unclear communication
94	Delays in surgery due to doctors strikes and additional medical issues.	Perceived Incompetence of Medical Management	Inadequate actions - delayed care
95	Feeling overlooked and dismissed by specialists despite ongoing severe pain and lack of effective treatment.	Disregard for Patient Concerns	Symptoms
96	Difficulty finding a doctor willing to prescribe treatment.	Other	Other
97	conflicting information from the GP and oncologist regarding the diagnosis	Communication	Conflicting information
98	Feeling rushed into chemotherapy without sufficient options or information.	Communication	Unclear communication
99	Concerns about out-of-network care and communication issues with oncologist.	Profit-Driven Medical Management	Other
100	lack of thorough testing and exploration of treatment options	Perceived Incompetence of Medical Management	Inadequate actions - perceived lack of thoroughness
101	Frustration with the centralized care system, poor communication, and issues with medication management.	Communication	Unclear communication
102	Unsure about the effectiveness of treatment and doubt in doctors' knowledge about disease progression.	Perceived Incompetence of Medical Management	Lack of knowledge/competency
103	Frustration with appointment delays and lack of timely information.	Perceived Incompetence of Medical Management	Inadequate actions - delayed care
104	Perceived lack of empathy and care from the surgeon during the follow-up appointment.	Communication	Perceived lack of empathy
105	Uncertainty about the test results and lack of clarity from the oncologist.	Communication	Unclear communication
106	concerns about side effects of prescribed medication	Other	Other
107	Doubts about the adequacy of the medical assessment regarding lymph node involvement	Lack of Trust in Medical Procedures	Diagnosis
108	Concern over interaction and effectiveness of medications prescribed.	Other	Other
109	Inconsistency in initial assessments about calcification and current concerns leading to worry.	Communication	Conflicting information

110	Concern that doctors may not take her pain and concerns seriously.	Disregard for Patient Concerns	Symptoms
111	Doubts about the oncologist's statement regarding curability despite evidence of possible metastasis.	Perceived Incompetence of Medical Management	Lack of knowledge/competency
112	Concern about the side effects of medication and possible lack of communication about risks involved.	Communication	Unclear communication
113	Delay in scheduling further treatment	Perceived Incompetence of Medical Management	Inadequate actions - delayed care
114	uncertainty regarding treatment decisions and communication from healthcare professional	Communication	Unclear communication
115	Perceived lack of adequate care and support from multiple healthcare providers, high costs of treatment, and feeling unsupported in managing health issues.	Disregard for Patient Concerns	Treatment wishes/expectations
116	Lack of prior information about the surgery details.	Communication	Unclear communication
117	Concern regarding potential side effects and effectiveness of prescribed medication.	Other	Other
118	The author feels that their partner is sharing personal medical information without considering their feelings, which they perceive as seeking sympathy rather than providing support.	Other	Other
119	Concerns about the length of wait times and the seeming delay in treatment.	Perceived Incompetence of Medical Management	Inadequate actions - delayed care
120	Concerns about missing clips and adequacy of the surgical margin.	Perceived Incompetence of Medical Management	Lack of knowledge/competency
121	Delays in the diagnosis and treatment process.	Perceived Incompetence of Medical Management	Inadequate actions - delayed care
122	Concerns over diagnostic errors and late detection of tumors.	Lack of Trust in Medical Procedures	Diagnosis
123	believed to be dishonest about treatment plan	Communication	Lack of Integrity
124	Concern over delays in treatment, unavailability of specialists, and overall resource scarcity.	Perceived Incompetence of Medical Management	Inadequate actions - delayed care
125	Concern over previous advice not to follow up on the cyst.	Perceived Incompetence of Medical Management	Inadequate actions - perceived lack of thoroughness
126	Inconsistent information about the presence of a mass and the effectiveness of treatment.	Communication	Conflicting information

127	Conflicting treatment recommendations and perceived lack of thoroughness in monitoring tumor growth.	Perceived Incompetence of Medical Management	Inadequate actions - perceived lack of thoroughness
128	The doctor does not take the patient seriously regarding their physical health issues.	Disregard for Patient Concerns	Other medical conditions
129	Belief that conventional treatments are inadequate or harmful.	Lack of Trust in Medical Procedures	Treatment
130	Concern about the harmful effects of chemotherapy on her mother's health.	Lack of Trust in Medical Procedures	Treatment
131	Concerns about honest communication regarding prognosis and potential misleading information from doctors.	Communication	Unclear communication
132	Poor communication, lack of effective diagnosis and treatment options from multiple healthcare professionals.	Communication	Unclear communication
133	Inconsistent information and unexpected pain during treatment.	Communication	Conflicting information
134	Long waiting time for critical diagnostic imaging and lack of communication regarding options.	Perceived Incompetence of Medical Management	Inadequate actions - delayed care
135	Perceived hesitance from doctors regarding treatment options.	Perceived Incompetence of Medical Management	Lack of knowledge/competency
136	Concerns about the speed of decline and effectiveness of care provided.	Perceived Incompetence of Medical Management	Treatment
137	Fear of potential diagnosis and previous experiences with anxiety.	Other	Other
138	Frustration with healthcare providers and the quality of care received.	Perceived Incompetence of Medical Management	Lack of knowledge/competency
139	Failure to suggest alternative methods to boost white blood cell count and communication issues regarding symptoms.	Disregard for Patient Concerns	Symptoms
140	Perceived lack of adequate pain management and follow-up from healthcare providers.	Perceived Incompetence of Medical Management	Inadequate actions - inadequate pain management
141	The feeling that the father's pain was dismissed and not adequately addressed by the healthcare system.	Disregard for Patient Concerns	Symptoms
142	Perceived inadequacies in pain management during hospice care.	Perceived Incompetence of Medical Management	Inadequate actions - inadequate pain management
143	Lack of available support during an emergency situation following a procedure.	Other	Other
144	Concern about limited treatment options after readmission.	Other	Other

145	Concerns about the decision to proceed with treatment despite not meeting the typical requirement.	Perceived Incompetence of Medical Management	Lack of knowledge/competency
146	concern about possible toxins causing cancer in both father and dog	Other	Other
147	perceived lack of timely communication from doctor and uncertainty about health outcomes	Communication	Unclear communication
148	perceived evasiveness of the healthcare provider regarding diagnostic information	Communication	Unclear communication
149	Concern that the doctor may be downplaying the vaccine side effects.	Communication	Lack of Integrity
150	Perceived lack of responsiveness and care from the healthcare team in critical health situations.	Communication	Lack of Integrity
151	Doctor's disapproval of medical marijuana and lack of openness in discussing options.	Disregard for Patient Concerns	Treatment wishes/expectations
152	The oncologist's suggestion to wait it out may seem dismissive given the author's new symptoms.	Disregard for Patient Concerns	Symptoms
153	Perception that the doctor downplayed the situation to ease the emotional impact.	Communication	Lack of Integrity
154	Feeling ignored and dismissed by the doctor regarding serious health concerns.	Disregard for Patient Concerns	Symptoms
155	carelessness and lack of support from close friends during a challenging time	Other	Other
156	The author feels dismissed by doctors, suggesting a belief that their concerns are not taken seriously.	Disregard for Patient Concerns	Other
157	Doubt about resolution of symptoms despite reassurances from oncologist	Disregard for Patient Concerns	Symptoms
158	Difficulty contacting the doctor for questions while she's out of the country.	Communication	Other
159	Concern about being referred to another doctor and feeling uncertain about the severity of the condition.	Communication	Unclear communication
160	Invalidation of cancer experience and lack of support from family and friends.	Other	Other
161	Belief that doctor may not be fully addressing the ongoing pain despite normal test results.	Disregard for Patient Concerns	Symptoms
162	Delayed communication and lack of information from doctors	Communication	Unclear communication
163	Frustration with unsolicited advice and the perceived lack of empathy from others.	Communication	Perceived lack of empathy
164	Feeling unsupported and judged by others who lack understanding of their experience.	Communication	Perceived lack of empathy
165	Plaintiff claims difficulties in receiving financial assistance from the charity.	Profit-Driven Medical Management	Other

166	Misunderstanding of symptoms and perceived dismissiveness regarding concerns.	Disregard for Patient Concerns	Symptoms
167	repeat delays in treatment	Perceived Incompetence of Medical Management	Inadequate actions - delayed care
168	Feeling of not being able to communicate directly with the doctors about the boyfriend's condition.	Communication	Unclear communication
169	Doctors not providing clear answers and only prescribing more tests.	Communication	Unclear communication

Supplementary Table 2

Supplementary Table 2: Exemplary posts (rephrased) containing mistrust by reason of mistrust category and subreddit.

Reason of mistrust	Subreddit	Post title (rephrased)	Post text (rephrased)
Communication	braincancer	Confused about brain tumour	I'm not sure where to post this, but my dad has a brain tumor that isn't cancerous. […] They are really leaving us in the dark about it no idea how big it has got to or anything.They aren't telling us anything, and I'm really worried.
Communication	coloncancer	Biopsy Results	My doctor initially said no follow-up was needed […] Today, I received a message to schedule an appointment to discuss the results. Does this imply cancer? I thought I would have been informed over the phone or via letter.
Communication	breastcancer	How to cope with isolation	[...] Now, my hands and feet are so sore and blistered that I can't do anything without hurting myself. [...] I tried to get help for my symptoms but was met with unprofessionalism. My day would have been better spent resting. [...] I just want to feel supported and understood.
Disregard for Patient Concerns	lymphoma	DLBCL on the basis of FL VS DLBCL	[...] I've read that DLBCL transformed from FL has a poorer prognosis, but I'm unsure if this means a poorer prognosis compared to non-transformed FL or primary DLBCL. My oncologist seems to dismiss the importance of my DLBCL being on the basis of FL. [...]
Disregard for Patient Concerns	coloncancer	UC seems more important than the Folfox actually working wrt surgery	[…] Tumor board and second opinion are pushing for massive surgery due to my ulcerative colitis, even though the UC isn't serious. They seem focused on it, which makes them think surgery is necessary. […]
Disregard for Patient Concerns	testicularcancer	I am still alive!!	[…] I hope most of you finishing chemo are improving. Personally, I feel like I'm getting worse in many areas, and my doctor is just being silly about it.
Lack of Trust in Medical Procedures	coloncancer	Colorectal cancer and overseas produced blood pressure drugs	My dad, who was diagnosed with stage 4 cancer, has been on Valsartan for nearly a decade. Interestingly, a couple of family friends who were prescribed Losartan also ended up being diagnosed with stage 4 colon cancer. I've heard there's a tort lawsuit linking some overseas-manufactured high blood pressure medications to colon cancer. [...] Has anyone else had experiences with high blood pressure medications?
Lack of Trust in Medical Procedures	thyroidcancer	Anyone have an Afirma benign result end up being cancerous in the end?	After reading about someone's Afirma benign result being wrong, I'm now wondering if my Afirma benign result can be trusted. They claim that a benign result indicates a 4% chance of cancer, but now I'm questioning whether that's accurate. […]
Lack of Trust in Medical Procedures	breastcancer	Radiotherapy for young breast cancer Patients	[...] My question is regarding radiation. I'm terrified of radiation because of potential future cancers, which are more likely to develop the longer I live. In half a century, I still won't be 80 years old. That's a long time to develop something like lung cancer or another breast cancer (I also have CHEK2). I also don't know that the research out there is really for someone my age. [...]
Perceived Incompetence of Medical Management	thyroidcancer	Your thoughts?	[...] I have a small 1.9 cm nodule, and the surgeon suggested a lobectomy without the need for radiation or further specialists. He doesn't think we need to radiate and biopsy this. This is different from what my primary care doctor expected. I'm considering a second opinion. [...]
Perceived	pancreaticca	Can You Start	[...] My mom has been dealing with a liver infection for the

Incompetence of Medical Management	ncer	chemotherapy with Signs of an Infection?	past 6–7 weeks. She’s been on both oral and IV antibiotics to treat it, but it’s taken this long because the doctors initially prescribed the wrong medication. At first, her oncologist said he’d be okay starting a reduced-dose chemo while the infection was improving. However, he’s since changed his stance and now insists the infection needs to be completely gone before proceeding. The issue is that we’re now more than three months post-surgery. The oncologist has mentioned he’s unsure if chemo will still be beneficial after four months, even though the research suggests otherwise. I very much dislike this oncologist, but unfortunately, we don’t have other options right now. [...]
Perceived Incompetence of Medical Management	pancreaticcancer	Need some help navigating this development.	[...] We visited Mayo Clinic, where the oncologist said the tumor is too complicated to operate on. Three biopsies later, all inconclusive but showing abnormal cells. Mayo is recommending chemo, but my dad is hesitant to start without a definitive diagnosis. [...] Has anyone had multiple inconclusive biopsies, especially at a place like Mayo, or started chemo without a confirmed diagnosis?
Profit-Driven Medical Management	thyroidcancer	Brand name synthroid is marked up literally 500% at retail price at my pharmacy. America's healthcare system is seriously messed up.	[...] $20 VERSUS $100?!?!?! That's literally a 500% increase of price, and I'm furious at the healthcare system. That’s a huge price difference! […]
Profit-Driven Medical Management	pancreaticcancer	My grandpa has pancreatic cancer and we are stuck.	[...] However, after 1.5 months, doctors couldn’t remove the cyst because it had grown three times larger. Now, he is extremely weak, barely able to sit, and is afraid of his heart. My family feels hopeless, and we think the hospital is just trying to make money off us. [...]
Profit-Driven Medical Management	breastcancer	Another rant from the newly diagnosed	[...] How could two tumors appear suddenly in my right breast, with no sign of them last year? Can I see the scans from last year for comparison?[...]It’s tough, and I feel like all our savings are going to medical bills, and our plans are slipping away. [...]
No mistrust	thyroidcancer	Nodule on thyroid bed?	Has anyone had a hypoechoic nodule found on an ultrasound after a thyroidectomy on the thyroid bed?
No mistrust	braincancer	MRI Help	Please help me interpret the MRI results. I'm 24 years old and experiencing several symptoms that I thought could be related to a blood clot or MS. […]
No mistrust	lymphoma	Tips for radiation?	[...] I’ve been told I may experience fatigue, swallowing issues, and a sore throat since they’ll be radiating my neck and chest. I’d love to hear about your experiences. What skin creams helped, and how did you manage fatigue and discomfort? […]

Supplementary Table 3

Supplementary Table 3: Frequency of Emotional Tones in the Exploratory Dataset as returned by the LLM, arranged by most frequent to least frequent term.

Number of occurrences	Terms
345	anxious
255	concerned
191	inquisitive
133	hopeful
90	worried
86	frustrated
85	uncertain
81	informative
79	supportive
76	curious
69	reflective
67	confused
58	frustration
40	seeking support
36	neutral
31	concern
29	fearful
28	sad
26	grateful
24	uncertainty,optimistic

23	anxiety
17	desperate
14	positive,humorous,confusion
13	scared,desperation
12	empathetic,thoughtful
10	sadness,vulnerable,distressed
9	inquiring,seeking help
7	informal,relieved,encouraging,hopeless,seeking advice,nervous
6	resilient,determined,sorrow,emotional,overwhelmed,devastated,light-hearted
5	apprehensive,exhausted,helpless,despair,conflicted
4	stressed,helplessness,grief,urgent,struggling,cautious,support-seeking,seeking information,upset,celebratory
3	skeptical,shock,unknown,casual,hope,guilty,sorrowful,seeking reassurance,disappointed,fear,nostalgic,vulnerability,unsure,caring
2	proactive,candid,cautionary,shocked,discouraged,anger,tired,help-seeking,reassured,anguished,sarcastic,angry,exhaustion,advocative,informational,heartbroken, cautiously optimistic,terrified,promotional,informed,depressed,inquiry,reassuring
1	accepting,thankful,polite,professional,generous,unclear,relief,motivational,suffering,trusting,hesitant,disassociated,advocacy,misleading,morbid,cynical,trust-seeking,reflection,guilt,business-oriented,bored,cautious optimism,sincere,clinical,unconventional,lonely,sensitive,uplifting,heartfelt,seeking validation,mourning,dejected,seeking guidance,engaged,requesting help,loss,content,empathy,peaceful,seeking,community-focused,community-oriented,request for information,acceptance,nostalgia,stress,emotionally charged,practical,worrisome,somber,humor,negative,querying,betrayal,panicked,wary,advisory, worry,hopelessness, hopeful,urgency,optimism,altruistic,searching,understanding,distrustful,self-reflective,suspicious,disbelief,distress,doubtful,accusatory,isolation,isolated,beseeching,curiosity,gratitude,gratefulness,sympathetic,appreciative,paranoid,frantic,disappointment,bizarre,calm, venting,insecurity,searching for help,pain,soothing,helpful,bewildered,annoyed,quest for information,brave,impatient,collaborative,requesting advice,bitter,spiritual,educational,despairing,slightly positive,doubt,seeking assistance,searching for support,resigned,serious,worrying,questioning,pessimistic

Supplementary Table 4

Supplementary Table 4: Frequency of Emotional Tones across mistrusted entities. Chi-squared test and Cramér's V were computed; p-values were adjusted using the Bonferroni correction.

Tone	Healthcare Institutions	Healthcare Professionals	Insurance Providers	Medical Science	Chi2.X-squared	Adjusted P value	Cramér's V
worried	912 (29.9)	4614 (32.4)	183 (22.2)	129 (27.6)	45.79341	<.0001	0.04966
concerned	545 (17.8)	2771 (19.5)	157 (19.1)	84 (17.9)	4.825874	>0.99	0.01612
anxious	426 (13.9)	2195 (15.4)	107 (13)	86 (18.4)	11.0658	0.67	0.02441
frustrated	587 (19.2)	1979 (13.9)	185 (22.5)	41 (8.8)	105.9246	<.0001	0.07553
inquisitive	269 (8.8)	1419 (10)	112 (13.6)	74 (15.8)	33.83916	<.0001	0.04269
supportive	78 (2.6)	255 (1.8)	25 (3)	5 (1.1)	14.6123	0.13	0.02805
confused	21 (0.7)	229 (1.6)	7 (0.9)	3 (0.6)	19.53075	0.01	0.03243
sad	58 (1.9)	193 (1.4)	2 (0.2)	6 (1.3)	13.77437	0.19	0.02724
hopeful	58 (1.9)	165 (1.2)	22 (2.7)	11 (2.4)	24.46403	0	0.0363
reflective	46 (1.5)	182 (1.3)	6 (0.7)	8 (1.7)	3.748215	>0.99	0.01421
informative	17 (0.6)	57 (0.4)	4 (0.5)	11 (2.4)	36.56525	<.0001	0.04438
uncertain	10 (0.3)	52 (0.4)	3 (0.4)	3 (0.6)	1.094553	>0.99	0.007678
seeking support	9 (0.3)	48 (0.3)	4 (0.5)	2 (0.4)	0.81221	>0.99	0.006614
fearful	10 (0.3)	40 (0.3)	3 (0.4)	2 (0.4)	0.610767	>0.99	0.005736
grateful	6 (0.2)	15 (0.1)	1 (0.1)	1 (0.2)	1.99498	>0.99	0.01037
curious	0 (0)	5 (0)	1 (0.1)	1 (0.2)	6.555075	>0.99	0.01879
optimistic	1 (0)	0 (0)	1 (0.1)	1 (0.2)	19.7729	0.01	0.03263
neutral	1 (0)	1 (0)	0 (0)	0 (0)	1.69257	>0.99	0.009548
concern	0 (0)	1 (0)	0 (0)	0 (0)	0.305551	>0.99	0.004057
frustration	0 (0)	0 (0)	0 (0)	0 (0)		NaN	

Supplementary Table 5

Supplementary Table 5: Frequency of Emotional Tones across reasons for mistrust. Chi-squared test and Cramér’s V were computed; p-values were adjusted using the Bonferroni correction.

Tone	Communication	Disregard Pt. Concerns	Lack of Trust in Med. Proc.	Perceived Incompetence	Profit Driven	Chi2.X-squared	Adjusted P value	Cramér's V
worried	1184 (29.2)	2556 (31.3)	253 (32.6)	1586 (32.6)	335 (27.2)	21.53926	0.01	0.03357
concerned	709 (17.5)	1568 (19.2)	159 (20.5)	927 (19)	253 (20.5)	9.396378	>0.99	0.02217
frustrated	523 (12.9)	1596 (19.5)	46 (5.9)	649 (13.3)	227 (18.4)	195.3079	<.0001	0.1011
anxious	796 (19.6)	1073 (13.1)	151 (19.5)	701 (14.4)	142 (11.5)	115.5542	<.0001	0.07775
inquisitive	517 (12.7)	672 (8.2)	107 (13.8)	448 (9.2)	168 (13.6)	96.56684	<.0001	0.07108
supportive	53 (1.3)	181 (2.2)	6 (0.8)	111 (2.3)	31 (2.5)	21.46354	0.02	0.03351
sad	27 (0.7)	112 (1.4)	7 (0.9)	134 (2.8)	6 (0.5)	82.31107	<.0001	0.06562
confused	111 (2.7)	81 (1)	7 (0.9)	59 (1.2)	4 (0.3)	76.68686	<.0001	0.06334
hopeful	45 (1.1)	96 (1.2)	12 (1.5)	76 (1.6)	32 (2.6)	19.66124	0.03	0.03207
reflective	38 (0.9)	108 (1.3)	9 (1.2)	87 (1.8)	12 (1)	13.89878	0.45	0.02697
informative	8 (0.2)	44 (0.5)	8 (1)	27 (0.6)	7 (0.6)	12.72864	0.75	0.02581
uncertain	24 (0.6)	19 (0.2)	2 (0.3)	20 (0.4)	3 (0.2)	10.9152	>0.99	0.0239
seeking support	13 (0.3)	27 (0.3)	1 (0.1)	19 (0.4)	5 (0.4)	1.605589	>0.99	0.009165
fearful	7 (0.2)	24 (0.3)	6 (0.8)	15 (0.3)	6 (0.5)	9.38331	>0.99	0.02216
grateful	2 (0)	11 (0.1)	1 (0.1)	9 (0.2)	0 (0)	5.015513	>0.99	0.0162
curious	1 (0)	5 (0.1)	0 (0)	1 (0)	0 (0)	2.583428	>0.99	0.01163
optimistic	1 (0)	0 (0)	0 (0)	1 (0)	1 (0.1)	5.049637	>0.99	0.01625
concern	0 (0)	1 (0)	0 (0)	1 (0)	0 (0)	1.131047	>0.99	0.007692
neutral	1 (0)	1 (0)	0 (0)	0 (0)	0 (0)	1.523009	>0.99	0.008926
frustration	0 (0)	1 (0)	0 (0)	0 (0)	0 (0)	1.337888	>0.99	0.008366

Supplementary Methods

Granular Categories for Reasons of Mistrust

1. Communication:
   - Unclear communication
   - Conflicting information
   - Perceived lack of empathy
   - Lack of Integrity
   - Other

2. Perceived Incompetence of Medical Management:
   - Lack of knowledge/competency
   - Inadequate actions - perceived lack of thoroughness
   - Inadequate actions - Inadequate pain management
   - Inadequate actions - Delayed care
   - Inadequate actions - Potential overtreatment
   - Inadequate actions - Misdiagnoses
   - Inadequate actions - Other
   - Other

3. Disregard for Patient Concerns:
   - Symptoms
   - Treatment wishes/expectations
   - Mental health problems
   - Other medical conditions
   - Other

4. Profit-Driven Medical Management:
   - By Insurance
   - By Health care providers
   - Other

5. Lack of Trust in Medical Procedures:
   - Treatment
   - Diagnosis
   - Lack of prioritization of cancer research
   - General diffuse mistrust of medical procedures
   - Other

6. Other

Prompts Used in this Study

1. Exploratory post analysis

```
Analyze the Provided Social Media Post Based on the Following Criteria:
   1.  Author Type: Identify the author as a patient, relative, healthcare professional, or other.
   2.  Cancer Type: Specify the cancer type discussed, or state “unknown”.
   3.  Sex: Specify the author's sex as male, female, or "unknown".
   4.  Disease Stage: Indicate the stage of the disease, or state “unknown”.
   5.  Tone: Analyze the primary tone of the post.
   6.  Depression Score: Assess the post for signs of depression and assign a score between 0 and 5.
   7.  Anxiety Score: Evaluate the anxiety level expressed in the post and assign a score between 0 and 5.
   8.  Distress Score: Evaluate the level of distress expressed in the post and assign a score between 0 and 5.
   10  Mistrust_Trust_NA: Evaluate whether the post reflects mistrust, trust, or lacks insufficient information to infer
trust/mistrust towards the healthcare system.
   - Definition: A belief that healthcare systems, institutions, or professionals may intentionally or unintentionally cause
harm, discriminate, or fail to act in the patient’s best interest.
   11. Object of distrust: The specific entity, individual, or system that is the focus of mistrust, if any.
   12. Reason for mistrust, if any.

Input Post:
{% include "post_template.j2" %}

Please analyze the post using the above criteria.
```

2. Main post analysis

```
Analyze the Provided Social Media Post Based on the Following Criteria:
   1.  Author Type: Identify the author as a patient, relative, healthcare professional, or other.
   2.  Cancer Type: Specify the cancer type discussed, or state “unknown”.
   3.  Sex: Specify the author's sex as male, female, or "unknown".
   4.  Disease Stage: Indicate the stage of the disease, or state “unknown”.
   5.  Tone: Analyze the primary tone of the post.
   6.  Depression Score: Assess the post for signs of depression and assign a score between 0 and 5.
   7.  Anxiety Score: Evaluate the anxiety level expressed in the post and assign a score between 0 and 5.
   8.  Distress Score: Evaluate the level of distress expressed in the post and assign a score between 0 and 5.
   10  Mistrust_Trust_NA: Evaluate whether the post reflects mistrust, trust, or lacks insufficient information to infer
trust/mistrust towards the healthcare system.
   - Definition: A belief that healthcare systems, institutions, or professionals may intentionally or unintentionally cause
harm, discriminate, or fail to act in the patient’s best interest.
```

```
   11. Object of distrust: The specific entity, individual, or system that is the focus of mistrust, if any.
   12. Reason for mistrust, if any.
   13. Object of mistrust category: Categorize the primary object of mistrust as Healthcare Professionals, Healthcare Institutions, Insurance Providers, Medical Science, or Other.
   14. Mistrust reason overall category: Categorize the primary reason for mistrust as one of the following:
      - Communication
      - Perceived Incompetence of Medical Management
      - Disregard for Patients Concerns
      - Profit-Driven Medical Management at the Expense of Patients
      - Lack of Trust in Medical Procedures in General

Input Post:
{% include "post_template.j2" %}

Please analyze the post using the above criteria.
```

3. Granular reason analysis

```
Analyze the Provided Social Media Post for Reasons of Mistrust regarding:
{{mistrust_reason_category}}

Input Post:
{% include "post_template.j2" %}

Analysis so far:
- Object of mistrust: {{object_of_mistrust}}
- Object of mistrust category: {{object_of_mistrust_category}}
- Mistrust reason: {{mistrust_reason}}
- Mistrust reason category: {{mistrust_reason_category}}

Please select the specific category:
{% include finegranlular_categories_filename %}


Please analyze the post using the criteria outlined above.
```

4. Post template

```
Post begin->:
**{{ title | safe }}**
{{ text  | safe}}
```

:<-Post end

Category definition

Human evaluators inductively derived the categories of mistrust used in this study and rated a sample of posts in order to determine the performance of LLM-based post classification. F.B., J.W., N.H. and M.S. participated in this evaluation process. All four evaluators were male, caucasian medical students, who passed their second state examination and had at least three years of prior research experience (basic or clinical research). At the time of the study, all evaluators were occupied with clinical rotations at a major cancer hospital in the US.

Large Language Models for Information Extraction

Large Language Models (LLMs) are artificial intelligence models designed to predict the next word in a sequence based on prior text[1]. This allows them to extract structured information by answering specific queries where the most probable next words serve as the extracted answer[2]. For example, consider the following hypothetical clinical note excerpt:

"During the consultation, the patient reported experiencing persistent fatigue, occasional headaches, and intermittent back pain over the past three weeks. The patient mentioned that the fatigue has been affecting daily activities and that the back pain sometimes worsens with physical exertion. No fever, cough, or other respiratory symptoms were noted during the evaluation."

If the model is then prompted, "What symptoms did the patient report? Please return the data in a structured format", the model processes the context and could extract the answer as follows:

```
{ "symptoms":
        [ "fatigue",
          "headache",
```

"back pain"]

}

This information can then be quantified, which allows the subsequent analyses to become more efficient, and possible on a large scale. Therefore, LLMs have been increasingly used for extracting structured data from unstructured text in various domains, including the retrieval of structured data from clinical notes and scientific texts, and sentiment analysis:

LLMs have been used to extract information from electronic health record (EHR) notes, for example admission time[3] as well as social determinants of health, such as educational status, smoking status, among others[4]. Further, LLMs have been used to extract psychopathological rating scores from EHR notes[5]. Structured information extraction was also explored regarding extraction from scientific literature, such as in the field of materials chemistry [2]. In online communities of epilepsy patients, LLMs have been used to extract topics discussed in social media posts [6].

While LLMs show strong performance in text extraction, their use comes with advantages and limitations. Their performance depends on the difficulty of the requested task, and the quality and structure of prompts used for extraction[7]. The performance of LLMs also depends on their training data, which can introduce biases[8]. Further, a lack of explainability in decision-making, and a risk of generating plausible but incorrect responses[9] have to be taken into consideration. Therefore, it is important to continuously evaluate their performance when applying them to extract information from large bodies of unstructured texts at scale.

For further details regarding LLMs in medicine, several comprehensive reviews [1, 10, 11] provide further insights into methodologies, challenges, and future directions.

References

1. Thirunavukarasu AJ, Ting DSJ, Elangovan K, Gutierrez L, Tan TF, Ting DSW. Large language models in medicine. Nat Med. 2023;29(8):1930-40.
2. Dagdelen J, Dunn A, Lee S, Walker N, Rosen AS, Ceder G, et al. Structured information extraction from scientific text with large language models. Nature communications. 2024;15(1):1418.
3. Lovon J, Mouysset M, Oleiwan J, Moreno JG, Damase-Michel C, Tamine L. Evaluating LLM Abilities to Understand Tabular Electronic Health Records: A Comprehensive Study of Patient Data Extraction and Retrieval. arXiv preprint arXiv:250109384. 2025.
4. Gu B, Shao V, Liao Z, Carducci V, Brufau SR, Yang J, et al. Scalable information extraction from free text electronic health records using large language models. BMC Med Res Methodol. 2025;25(1):23.
5. McCoy TH, Jr., Perlis RH. Dimensional Measures of Psychopathology in Children and Adolescents Using Large Language Models. Biol Psychiatry. 2024;96(12):940-7.
6. Fennig U, Yom-Tov E, Savitsky L, Nissan J, Altman K, Loebenstein R, et al. Bridging the conversational gap in epilepsy: Using large language models to reveal insights into patient behavior and concerns from online discussions. Epilepsia. 2024.
7. Cao B, Cai D, Zhang Z, Zou Y, Lam W. On the worst prompt performance of large language models. Advances in Neural Information Processing Systems. 2025;37:69022-42.
8. Prakash N, Roy LKW. Interpreting Bias in Large Language Models: A Feature-Based Approach. arXiv preprint arXiv:240612347. 2024.
9. Sarkar A. Large Language Models Cannot Explain Themselves. arXiv preprint arXiv:240504382. 2024.
10. Clusmann J, Kolbinger FR, Muti HS, Carrero ZI, Eckardt JN, Laleh NG, et al. The future landscape of large language models in medicine. Commun Med (Lond). 2023;3(1):141.
11. Omiye JA, Gui H, Rezaei SJ, Zou J, Daneshjou R. Large Language Models in Medicine: The Potentials and Pitfalls : A Narrative Review. Ann Intern Med. 2024;177(2):210-20.
